# Interval-valued intuitionistic fuzzy rough set system over a novel conflict distance measure with application to decision-making

**DOI:** 10.1016/j.mex.2023.102012

**Published:** 2023-01-13

**Authors:** Ashutosh Tiwari, Q.M. Danish Lohani

**Affiliations:** Department of Mathematics, South Asian University, New Delhi 110021, India

**Keywords:** Interval-valued intuitionistic fuzzy sets, Conflict analysis, Conflict distance, Rough sets, Interval-valued intuitionistic fuzzy rough set system over a novel conflict distance measure

## Abstract

Conflict analysis is one of the most critical application domains whose importance is increasing rapidly nowadays. Attributes involving conflicts frequently occur with opinion, negotiations, and collaborators in decision-making. Taking advantage of the uncertainty present in decision-making, in this paper, we have proposed a system that can solve the problems involving conflicts more adequately.

• A new interval-valued intuitionistic fuzzy rough set (IVIFRS) system is introduced to handle a decision-making problem involving a conflict of interests.
• The proposed system exploits both the notions of rough set and interval-valued intuitionistic fuzzy set theories in sharpening the boundaries of conflicts.
• In the IVIFRS system, the disputes amongst the objectives are measured by the novel conflict distance measure. Further, an interval-valued intuitionistic fuzzy conflict analysis system formulated on the IVIFRS is designed for deciding the conflicting attributes

The formulated system is then studied for weight vectors too. The intended conflict analysis system is studied with reference to the well-known existing intuitionistic fuzzy rough set system. The real-life socio-economic problems are dealt with, and the experimental results validate the efficacy of the proposed system.

## Graphical abstract

**Figure:**
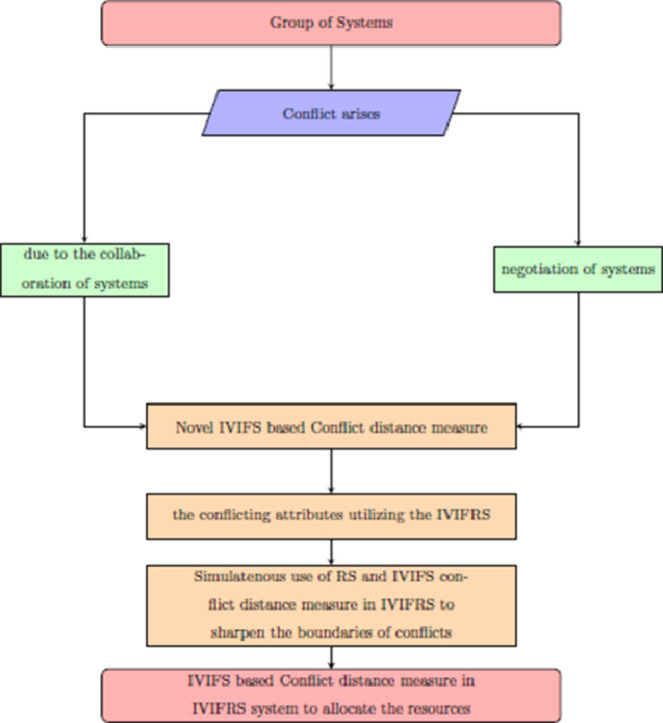

Specifications Table

**Table:** 

Subject Area	Mathematics
More specific subject area	Intuitionistic Fuzzy Set Theory
Method name	Interval-valued intuitionistic fuzzy rough set system over a novel conflict distance measure
Name and reference of original method	Intuitionistic fuzzy rough set model based on conflict distance. https://www.sciencedirect.com/science/article/pii/S1568494615001672
Resource availability	N/A

## Introduction

An interval-valued intuitionistic fuzzy set (IVIFS) is the bridge that connects Atanassov intuitionistic fuzzy set (AIFS) and interval-valued fuzzy set (IVFS). It is proposed by Atanassov, and Gargov [1]. The IVIFS contains much uncertain information as it expresses the information regarding membership and non-membership intervals. In other words, it is a blend that naturally extends fuzzy set [2], IVFS [3], and AIFS [4]. Only membership values are assigned to the elements in a fuzzy set, whereas IVFS gives the membership interval. The membership and non-membership values are used to define an AIFS. The IVIFS differs from AIFS theoretically, but this difference can not be analyzed mathematically [5]. AIFS is a very popular generalization of fuzzy set, and it has an extensive range of applications such as decision-making [6], [7], health sector [8], [9], economics [10], [11] etc. Usually, the membership and non-membership functions of AIFS contain parameters, and the values of the parameters are selected from intervals. In some cases, the membership and non-membership functions of AIFS are not precisely driven, so the only available option is to approximate them in some range. This approximate estimation yields IVIFS. The operators over IVIFS are defined by Atanassov [12], Burillo and Bustince [13], and Xu [14], [15]. In recent years, The practical application of IVIFS has been shown in the fields of decision-making [5], [16], [17] and pattern recognition [18], [19], [20], etc. Mondal and Samanta [21] studied the topology of the IVIFS. In the application domain, the IVIFS is not explored much. The paper shows IVIFS as a supplement to the resource allocation problem.

The equivalence relation is used to introduce the rough set (RS), and it involves lower and upper approximation of elements [22]. The IVIFS is defined with the help of an upper and lower approximation. So, it is quite interesting to study the interrelationship between RS and IVIFS. This interrelationship is being discussed while solving some decision-making problems. The universe of discourse of decision-making problems needs to be divided into equivalence classes for decision-making. It is difficult to define the equivalence relation as per the requirement of the problem, so upper and lower approximations-based relations used in a rough set help in modeling the problems. The IVIFS is used to extract the upper and lower approximations in the paper. The three categories of decisions are observed while dealing with the problem with the help of RS, namely positive, negative and uncategorized.

In the AIFS domain, several researchers explored the concept of RS (see: [23], Nanda and Majumdar [24], Chakrabarty et al. [25], Jenna et al. [26], [27], Zhou et al. [28], Huang et al. [29], Zhang [30]). Recently, a mathematical equivalence has been established between AIFS and IVIFS [5]. Some knowledge reduction and rule extraction techniques use classical and generalized RS theories (see: [31], [32], [33]). The IVIFS and RS are also simultaneously used in the proposal of knowledge reduction technique [34], dominance-based interval-valued intuitionistic fuzzy information system [35], and the interval-valued intuitionistic fuzzy rough set (IVIFRS) approximation operators and relations [30]. In the application domain, the simultaneous use of IVIFS and RS solved problems of pattern recognition, medical sciences [36], [37], [38], [39], and conflict analysis [40]. In decision-making, we often see that the similarity/distance measure introduced on merging IVIFS and RS results in good decisions [41], [42], [43]. A conflict is observed within the membership and non-membership values of IVIFS/AIFS, and many distance/similarity measures do not account for this conflict.

The conflicting situation can be interpreted in terms of three binary relations: alliance (coalition/favorable), against (conflict), and neutrality [44] between agents. The primary concern in a conflict situation is appropriately modeling the uncertainty [45], [46], [47]. The hesitancy interval/value of IVIFS/AIFS is exploited to analyze the conflict [48], [49], [50]. In addition to the hesitancy interval of the element, the researchers used the favor and opposition degree of elements to assign a score value to the element. The score function-based distance/similarity measures model the conflicting environment. Liu and Lin [40] used an intuitionistic fuzzy rough set to explore the conflict measure. They used a score function that could not differentiate between some intuitionistic fuzzy numbers (IFNs). Still, several conflicting decision-making problems are handled using IVIFS and RS theories. Conflict analysis is used in economics, business, governmental policies, political disputes, management negotiations, and military operations [51], [52], [53].

Hence, a novel decision-making system is proposed in which IVIFS and RS are being used. Further, a score function-based distance measure for conflicting situations is introduced. Here, the positive and negative approximations of RS are evaluated using IVIFS. The proposed decision-making system is an interval-valued intuitionistic fuzzy information (IVIFRS) system. The proposed IVIFRS system is modified as per the requirement of conflict analysis. The contributions of the paper are as follows:

1. A score function-based conflict distance measure is proposed over the interval-valued intuitionistic fuzzy environment.
2. A novel IVIFRS system is introduced by blending the theories of IVIFS and RS.
3. A modified IVIFRS system is proposed to deal with the conflicting situation.
4. In the paper, mathematical and analytical validation of the modified IVIFRS system is carried out. Further, the coalition between the elements is also accessed by the modified IVIFRS.
5. The modified IVIFRS system works on the two different decision-making problems. There is a reduction in the real conflict of the entire system.

The remaining paper proceeds this way; Section “Basic concepts” presents the basic definitions of IFS, IVIFS, and RS theories. Section “Proposed interval-valued intutionistic fuzzy information system based on the novel conflict distance measure” pretenses the IVIFRS system based on the proposed conflict distance measure. Section “IVIFIS based conflict analysis system” put forward an interval-valued intuitionistic fuzzy conflict analysis system. Section “Rough set” contains the studies and experimental results obtained using the proposed approach. At last, the conclusion and future directions are in Section “Case study”.

## Basic concepts

This section contains the basic definitions useful for the understanding of the paper.

Definition 1 ***Interval-valued intuitionistic fuzzy set***[1]

Let X be a universe of discourse. We define a set A as follows:

(1) $$A=\{(x,\left[\omega_{A}^{L}\left(x\right),\omega_{A}^{U}\left(x\right)\right],\left[\upsilon_{A}^{L}\left(x\right),\upsilon_{A}^{U}\left(x\right)\right])|x\in X\},$$

where, $\left[\omega_{A}^{L}\left(x\right),\omega_{A}^{U}\left(x\right)\right]\in I\left(\left[0,1\right]\right)$ and $\left[\upsilon_{A}^{L}\left(x\right),\upsilon_{A}^{U}\left(x\right)\right]\in I\left(\left[0,1\right]\right)$, such that $0\leq \omega_{A}^{U}\left(x\right)+\upsilon_{A}^{U}\left(x\right)\leq 1$, $0\leq \omega_{A}^{L}\left(x\right)+\upsilon_{A}^{L}\left(x\right)\leq 1$. The symbol I([0,1]) denotes a closed intervl that lies within [0,1]. This set A is an IVIFS. Here, $\left[\omega_{A}^{L}\left(x\right),\omega_{A}^{U}\left(x\right)\right]$ and $\left[\upsilon_{A}^{L}\left(x\right),\upsilon_{A}^{U}\left(x\right)\right]$ are the membership interval and non-membership interval of the IVIFS, respectively. There exist another interval $\left[\pi_{A}^{L}\left(x\right),\pi_{A}^{U}\left(x\right)\right]$ in IVIFS known as hesitancy interval, with $\pi_{A}^{L}\left(x\right)=1-\omega_{A}^{U}\left(x\right)-\upsilon_{A}^{U}\left(x\right)$ and $\pi_{A}^{U}\left(x\right)=1-\omega_{A}^{L}\left(x\right)-\upsilon_{A}^{L}\left(x\right)$.

The interval-valued intuitionistic fuzzy number (IVIFN) based on the IVIFS is $\left(\left[\omega_{A}^{L}\left(x\right),\omega_{A}^{U}\left(x\right)\right],\left[\upsilon_{A}^{L}\left(x\right),\upsilon_{A}^{U}\left(x\right)\right]\right)$ [12] and we represent it as $\tilde{a}=(\left[\omega^{L},\omega^{U}\right],\left[\upsilon^{L},\upsilon^{U}\right])$.

Definition 2 ***Algebraic Operations between IVIFNs***[54]

Let $\tilde{a},\tilde{b},\tilde{c}$ be the given IVIFNs, such that $\tilde{a}=(\left[\omega_{1}^{L},\omega_{1}^{U}\right],\left[\upsilon_{1}^{L},\upsilon_{1}^{U}\right])$, $\tilde{b}=(\left[\omega_{2}^{L},\omega_{2}^{U}\right],\left[\upsilon_{2}^{L},\upsilon_{2}^{U}\right])$ and $\tilde{c}=(\left[\omega^{L},\omega^{U}\right],\left[\upsilon^{L},\upsilon^{U}\right])$ and κ>0. Now,

(1) $\tilde{a}⊕\tilde{b}=(\left[\omega_{1}^{L}+\omega_{2}^{L}-\omega_{1}^{L}\omega_{2}^{L},\omega_{1}^{U}+\omega_{2}^{U}-\omega_{1}^{U}\omega_{2}^{U}\right],\left[\upsilon_{1}^{L}\upsilon_{2}^{L},\upsilon_{1}^{U}\upsilon_{2}^{U}\right])$
(2) $\tilde{a}⊖\tilde{b}\;=\;\{\left[min\left(\omega_{1}^{L},\omega_{2}^{L}\right),min\left(\omega_{1}^{U},\omega_{2}^{U}\right)\right],\left[max\left(\upsilon_{1}^{L},\upsilon_{2}^{L}\right),max\left(\upsilon_{1}^{U},\upsilon_{2}^{U}\right)\right]\}$
(3) $\tilde{a}⊗\tilde{b}=(\left[\omega_{1}^{L}\omega_{2}^{L},\omega_{1}^{U}\omega_{2}^{U}\right],\left[\upsilon_{1}^{L}+\upsilon_{2}^{L}-\upsilon_{1}^{L}\upsilon_{2}^{L},\upsilon_{1}^{U}+\upsilon_{2}^{U}-\upsilon_{1}^{U}\upsilon_{2}^{U}\right])$
(4) $\kappa \tilde{c}=(\left[1-{\left(1-\omega^{L}\right)}^{\kappa },1-{\left(1-\omega^{U}\right)}^{\kappa }\right],\left[{\left(\upsilon^{L}\right)}^{\kappa },{\left(\upsilon^{U}\right)}^{\kappa }\right])$
(5) ${\tilde{c}}^{\kappa }=(\left[{\left(\omega^{L}\right)}^{\kappa },{\left(\omega^{U}\right)}^{\kappa }\right)],\left[1-{\left(1-\upsilon^{L}\right)}^{\kappa },1-{\left(1-\upsilon^{U}\right)}^{\kappa }\right])$

Definition 3 ***Score function and Accuracy function***[14]

Let $\tilde{a}=(\left[\omega^{L},\omega^{U}\right],\left[\upsilon^{L},\upsilon^{U}\right])$ be an IVIFN. The score function $S(\tilde{a})$ is given as follows:

(2) $$S\left(\tilde{a}\right)=\frac{\left(\omega^{L}-\upsilon^{L}\right)+\left(\omega^{U}-\upsilon^{U}\right)}{2},\;\;S\left(\tilde{a}\right)\in \left[-1,1\right]$$

and the accuracy function $H(\tilde{a})$ is defined as follows:

(3) $$H\left(\tilde{a}\right)=\frac{\omega^{L}+\upsilon^{L}+\omega^{U}+\upsilon^{U}}{2},\;\;H\left(\tilde{a}\right)\in \left[0,1\right]$$

The score function measures the size of $S(\tilde{a})$, whereas its accuracy is determined by the $H(\tilde{a})$. Larger value of score function implies bigger is the IVIFN, and larger value of accuracy function implies more accurate is the IVIFN.

Proposition 1 *Interrelationship between Score functions and IVIFNs**[14]*

*Let*$\tilde{a}=(\left[\omega_{1}^{L},\omega_{1}^{U}\right],\left[\upsilon_{1}^{L},\upsilon_{1}^{U}\right])$*,*$\tilde{b}=(\left[\omega_{2}^{L},\omega_{2}^{U}\right],\left[\upsilon_{2}^{L},\upsilon_{2}^{U}\right])$*are two IVIFNs. Then we have the following:*

(1) *If*$S\left(\tilde{a}\right)<S\left(\tilde{b}\right)$*then*$\tilde{a}<\tilde{b}$
(2) *If*$S\left(\tilde{a}\right)>S\left(\tilde{b}\right)$*then*$\tilde{a}>\tilde{b}$
(3) *If*$S\left(\tilde{a}\right)=S\left(\tilde{b}\right)$*, then accuracy function is used for comparison of*$\tilde{a}$*and*$\tilde{b}$*in following manner:*
  (i) *If*$H\left(\tilde{a}\right)>H\left(\tilde{b}\right)$*then*$\tilde{a}>\tilde{b}$*,*
  (ii) *If*$H\left(\tilde{a}\right)>H\left(\tilde{b}\right)$*then*$\tilde{a}>\tilde{b}$*,*
  (iii) *If*$H\left(\tilde{a}\right)=H\left(\tilde{b}\right)$*then*$\tilde{a}$≈$\tilde{b}$

Definition 4 ***Improved score function***[55]

Let us consider an IVIFN $\tilde{a}=(\left[\omega^{L},\omega^{U}\right],\left[\upsilon^{L},\upsilon^{U}\right])$, which satisfies the following properties:

(1) There exist a hesitancy interval $\left[1-\omega^{U}-\upsilon^{U},1-\omega^{L}-\upsilon^{L}\right]$ for $\tilde{a}$
(2) There exist a favorable interval $\left[\omega^{L}+\omega^{L}\left(1-\omega^{U}-\upsilon^{U}\right),\omega^{U}+\omega^{U}\left(1-\omega^{L}-\upsilon^{L}\right)\right]$ for $\tilde{a}$
(3) There exist an opposition interval $\left[\upsilon^{L}+\upsilon^{L}\left(1-\omega^{U}-\upsilon^{U}\right),\upsilon^{U}+\upsilon^{U}\left(1-\omega^{L}-\upsilon^{L}\right)\right]$ for $\tilde{a}$

The improved score function $S_{1}\left(\tilde{a}\right)$ is of the following form:

(4) $$S_{1}\left(\tilde{a}\right)=\frac{\left(\omega^{L}-\upsilon^{L}\right)\left(2-\omega^{L}-\upsilon^{L}\right)+\left(\omega^{U}-\upsilon^{U}\right)\left(2-\omega^{U}-\upsilon^{U}\right)}{2},\;\;S_{1}\left(\tilde{a}\right)\in \left[-1,1\right]$$

Definition 5 ***Distance Measure between IVIFSs***[56]

Let D:IVIFS(X)×IVIFS(X) → [0,1] such that it satisfies the following properties where $A_{1}$ and $A_{2}$ are IVIFS,

(1) $0\leq D(A_{1},A_{2})\leq 1$
(2) $D(A_{1},A_{2})$ = 0 if and only if $A_{1}$ = $A_{2}$
(3) $D(A_{1},A_{2})$ = $D(A_{2},A_{1})$
(4) If $A_{1}\subseteq A_{2}\subseteq A_{3}$, where $A_{1},A_{2},A_{3}$ ∈ IVIFSs(X), then $D(A_{1},A_{3})$ ≥ $D(A_{1},A_{2})$ and $D(A_{1},A_{3})$ ≥ $D(A_{2},A_{3})$

The similarity measure corresponding to distance measure is formulated in [57] which is:

(5) $$s\left(A_{1},A_{2}\right)=1-D\left(A_{1},A_{2}\right).$$

Definition 6 ***Rough Set***[22]

The pair (X,R) is Pawlak approximation space with X as a universe of discourse, and R as an equivalence relation defined on U. A partition X/R={[x]R:x∈X} on X will be generated by R, where [x]R is the equivalence class regarding R containing x. For every U⊆X, the lower approximation $\underline{R(U)}$ and upper approximation $\bar{R(U)}$ of U with respect to (X,R) is described as:

(6) $$\underline{R(U)}=\left\{x\in X:\left[x\right]R\subseteq U\right\}\;\;\text{and}\;\;\bar{R(U)}=\left\{x\in X:\left[x\right]R\cap U\neq \phi \right\}$$

is termed definable in (X,R) if $\underline{R(U)}=\bar{R(U)}$, or else U is known as rough set.

## Proposed interval-valued intutionistic fuzzy information system based on the novel conflict distance measure

Let X be a given universe of discourse. We have conditional attributes, say $A=\{a_{1},a_{2},\ldots ,a_{p}\}$, and decision attribute, say $D=\{d_{1},d_{2},\ldots ,d_{m}\}$, such that A∩D=∅. Let us define a map f:X×(A∪D)→Y, where Y is the collection of all the conditional and decision attributes. A quadruple Q=(X,A∪D,Y,f) is called an interval-valued intuitionistic fuzzy information system (IVIFIS) if the information is attached with $f\left(x,a_{i}\right)\in Y,\;\forall \;a_{i}\in A\cup D,\left(i=1,2,\ldots ,p\right)$. The information is delivered in terms of IVIFNs, so $f\left(x,a_{i}\right)=(\left[\omega_{a_{i}}^{L}\left(x\right),\omega_{a_{i}}^{U}\left(x\right)\right],\left[\upsilon_{a_{i}}^{L}\left(x\right),\upsilon_{a_{i}}^{U}\left(x\right)\right])$. Here, IVIFNs derive the upper approximation and lower approximations of rough sets.

Yong and Yi [40] have shown the advantage of using a rough set in an intuitionistic fuzzy environment while solving a conflict problem. The score function used in [40] could not differentiate some of the IVIFNs given in the paper. Here, $\tilde{a}\approx \tilde{b}$ symbolizes that $\tilde{a}\;\text{and}\;\tilde{b}$ are not comparable, as per the def. (2.3-2). We select two IVIFNs, $\tilde{a}=(\left[0.2,0.6\right],\left[0.2,0.4\right])$ and $\tilde{b}=(\left[0.3,0.5\right],\left[0.1,0.5\right])$. Now, $S\left(\tilde{a}\right)=S\left(\tilde{b}\right)=0.1$ and $H\left(\tilde{a}\right)=H\left(\tilde{b}\right)=0.7$, and hence, S and H fails to differentiate between $\tilde{a}\;\text{and}\;\tilde{b}$. It has motivated researchers to propose another type of score function. The improved score function (see: def. 4), results $S_{1}\left(\tilde{a}\right)=0.10$ and $S_{1}\left(\tilde{b}\right)=0.16$, which implies $\tilde{a}<\tilde{b}$. Hence, the improved score function is capable of distinguishing IVIFNs $\tilde{a}\;\text{and}\;\tilde{b}$. So, the paper proposes a new IVIFS conflict distance measure using an improved score function. We have discussed the role of the core, support, and hesitancy (see: def. 4) in the proposed conflict distance measure.

Now our conflict distance measure is defined as follows:

Let (X,A∪D,Y,f) be a given IVIFIS. Now, we define a conflict distance $\zeta_{xy}^{t}$ for IVIFIS, where x,y∈X, t∈T and T⊆A, as follows:

(7) $$\begin{array}{ccc} & & \zeta_{xy}^{t}=\frac{1}{8}\left(\right|S_{x}^{t}\left(a_{x}\right)-S_{y}^{t}\left(a_{y}\right)\left|+\right|\Delta_{x}^{t}\left(a_{x}\right)-\Delta_{y}^{t}\left(a_{y}\right)\left|+\right|\rho_{x}^{t}\left(a_{x}\right)-\rho_{y}^{t}\left(a_{y}\right)\left|\right)\\ & & \text{where,}\;a_{x}^{t}=(\left[\omega_{x}^{tL},\omega_{x}^{tU}\right],\left[\upsilon_{x}^{tL},\upsilon_{x}^{tU}\right]),\;\;a_{y}^{t}=(\left[\omega_{y}^{tL},\omega_{y}^{tU}\right],\left[\upsilon_{y}^{tL},\upsilon_{y}^{tU}\right]),\\ & & \;\;\;\;\;\;\;\;\;\;\pi_{x}^{t}=\left[\pi_{x}^{tL},\pi_{x}^{tU}\right],\pi_{y}^{t}=\left[\pi_{y}^{tL},\pi_{y}^{tU}\right];\\ & & S_{x}^{t}\left(a_{x}\right)=(\omega_{x}^{tL}-\upsilon_{x}^{tL})\left(1+\pi_{x}^{tU}\right)+\left(\omega_{x}^{tU}-\upsilon_{x}^{tU}\right)\left(1+\pi_{x}^{tL}\right)),\\ & & \;S_{y}^{t}\left(a_{y}\right)=(\omega_{y}^{tL}-\upsilon_{y}^{tL})\left(1+\pi_{y}^{tU}\right)+\left(\omega_{y}^{tU}-\upsilon_{y}^{tU}\right)\left(1+\pi_{y}^{tL}\right));\\ & & \pi_{x}^{t}=(1-\omega_{x}^{tU}-\upsilon_{x}^{tU},1-\omega_{x}^{tL}-\upsilon_{x}^{tL}),\;\;\pi_{y}^{t}=(1-\omega_{y}^{tU}-\upsilon_{y}^{tU},1-\omega_{y}^{tL}-\upsilon_{y}^{tL});\\ & & \Delta_{x}^{t}=(\omega_{x}^{tL}+\omega_{x}^{tL}\pi_{x}^{tU}+\omega_{x}^{tU}+\omega_{x}^{tU}\pi_{x}^{tL}),\;\;\Delta_{y}^{t}=(\omega_{y}^{tL}+\omega_{y}^{tL}\pi_{y}^{tU}+\omega_{y}^{tU}+\omega_{y}^{tU}\pi_{y}^{tL});\\ & & \rho_{x}^{t}=(\upsilon_{x}^{tL}+\upsilon_{x}^{tL}\pi_{x}^{tU}+\upsilon_{x}^{tU}+\upsilon_{x}^{tU}\pi_{x}^{tL}),\;\;\rho_{y}^{t}=(\upsilon_{y}^{tL}+\upsilon_{y}^{tL}\pi_{y}^{tU}+\upsilon_{y}^{tU}+\upsilon_{y}^{tU}\pi_{y}^{tL}). \end{array}$$

Remark 1 In IVIFS, the lower and upper approximations of (μ,ν,π) exist within the AIFS domain. Thus, the rough set is not involved in defining our IVIFIS. Moreover, the proposal of conflict distance measure is sufficient to introduce the IVIFIS.

Remark 2 Researchers define score, favor, and opposition [58], [59]. In the conflict distance measure (see: Eq. (7)), instead of the deviations of the membership, non-membership, and hesitancy intervals, the score, favor, and opposition are used. Here, the score compares the IVIFNs. Two indices, namely favor and opposition provide a critical view of membership and non-membership.

Now, we validate mathematically that the proposed conflict distance measure $\zeta_{xy}^{t}$ satisfies all the axioms of the distance measure.

Lemma 1 $\zeta_{xx}^{l}=0,\;\zeta_{xx}^{A}=0.$

Proof As per the Eq. (7) and $\zeta_{xy}^{l}=\sum_{l=1}^{p}w_{l}\zeta_{xy}^{l}$, it is obvious that $\zeta_{xx}^{l}=0$ and then $\zeta_{xx}^{A}=0$. □

Property 1 $\;0\leq \zeta_{xy}^{l}\leq 1$*,*$\;0\leq \zeta_{xy}^{A}\leq 1.$

Proof According to new conflict distance on IVIFIS, we have:

$$\zeta_{xy}^{l}=\frac{1}{8}\left(\right|S_{x}^{l}\left(a_{x}\right)-S_{y}^{l}\left(a_{y}\right)\left|+\right|\Delta_{x}^{l}\left(a_{x}\right)-\Delta_{y}^{l}\left(a_{y}\right)\left|+\right|\rho_{x}^{l}\left(a_{x}\right)-\rho_{y}^{l}\left(a_{y}\right)\left|\right)$$

with, $a_{x}^{l}=(\left[\omega_{x}^{lL},\omega_{x}^{lU}\right],\left[\upsilon_{x}^{lL},\upsilon_{x}^{lU}\right])$,  $a_{y}^{l}=(\left[\omega_{y}^{lL},\omega_{y}^{lU}\right],\left[\upsilon_{y}^{lL},\upsilon_{y}^{lU}\right]),\;\pi_{x}^{l}=\left[\pi_{x}^{lL},\pi_{x}^{lU}\right]$

$\;\;\;\;\;\;\pi_{y}^{l}=\left[\pi_{y}^{lL},\pi_{y}^{lU}\right]$;

$\;\;\;\;S_{x}^{l}\left(a_{x}\right)=(\omega_{x}^{lL}-\upsilon_{x}^{lL})\left(1+\pi_{x}^{lU}\right)+\left(\omega_{x}^{lU}-\upsilon_{x}^{lU}\right)\left(1+\pi_{x}^{lL}\right))$,

$\;\;\;\;S_{y}^{l}\left(a_{y}\right)=(\omega_{y}^{lL}-\upsilon_{y}^{lL})\left(1+\pi_{y}^{lU}\right)+\left(\omega_{y}^{lU}-\upsilon_{y}^{lU}\right)\left(1+\pi_{y}^{lL}\right))$;

$\;\;\;\;\pi_{x}^{l}=(1-\omega_{x}^{lU}-\upsilon_{x}^{lU},1-\omega_{x}^{lL}-\upsilon_{x}^{lL})$, $\;\;\pi_{y}^{t}=(1-\omega_{y}^{tU}-\upsilon_{y}^{tU},1-\omega_{y}^{tL}-\upsilon_{y}^{tL})$;

$\;\;\;\;\Delta_{x}^{l}=(\omega_{x}^{lL}+\omega_{x}^{lL}\pi_{x}^{lU}+\omega_{x}^{lU}+\omega_{x}^{lU}\pi_{x}^{lL})$, $\;\;\Delta_{y}^{l}=(\omega_{y}^{lL}+\omega_{y}^{lL}\pi_{y}^{lU}+\omega_{y}^{lU}+\omega_{y}^{lU}\pi_{y}^{lL})$;

$\;\;\;\;\rho_{x}^{l}=(\upsilon_{x}^{lL}+\upsilon_{x}^{lL}\pi_{x}^{lU}+\upsilon_{x}^{lU}+\upsilon_{x}^{lU}\pi_{x}^{lL})$, $\;\;\rho_{y}^{l}=(\upsilon_{y}^{lL}+\upsilon_{y}^{lL}\pi_{y}^{lU}+\upsilon_{y}^{lU}+\upsilon_{y}^{lU}\pi_{y}^{lL})$.

$\;\;\;0\leq \omega_{x}^{lL}+\upsilon_{x}^{lL}\leq 1,\;0\leq \omega_{x}^{lU}+\upsilon_{x}^{lU}\leq 1;\;\;0\leq \omega_{y}^{lL}+\upsilon_{y}^{lL}\leq 1,\;0\leq \omega_{y}^{lU}+\upsilon_{y}^{lU}\leq 1.$ putting above mentioned values in expression,

$$\left(\right|S_{x}^{l}\left(a_{x}\right)-S_{y}^{l}\left(a_{y}\right)\left|+\right|\Delta_{x}^{l}\left(a_{x}\right)-\Delta_{y}^{l}\left(a_{y}\right)\left|+\right|\rho_{x}^{l}\left(a_{x}\right)-\rho_{y}^{l}\left(a_{y}\right)\left|\right)$$

we have,

$$\begin{array}{ccc} & & |((\omega_{x}^{lL}-\upsilon_{x}^{lL})\left(1+\pi_{x}^{lU}\right)+\left(\omega_{x}^{lU}-\upsilon_{x}^{lU}\right)\left(1+\pi_{x}^{lL}\right))-((\omega_{y}^{lL}-\upsilon_{y}^{lL})\left(1+\pi_{y}^{lU}\right)+\left(\omega_{y}^{lU}-\upsilon_{y}^{lU}\right)\left(1+\pi_{y}^{lL}\right))|\\ & & +|(\omega_{x}^{lL}+\omega_{x}^{lL}\pi_{x}^{lU}+\omega_{x}^{lU}+\omega_{x}^{lU}\pi_{x}^{lL})-(\omega_{y}^{lL}+\omega_{y}^{lL}\pi_{y}^{lU}+\omega_{y}^{lU}+\omega_{y}^{lU}\pi_{y}^{lL})|\\ & & +|(\upsilon_{x}^{lL}+\upsilon_{x}^{lL}\pi_{x}^{lU}+\upsilon_{x}^{lU}+\upsilon_{x}^{lU}\pi_{x}^{lL})-(\upsilon_{y}^{lL}+\upsilon_{y}^{lL}\pi_{y}^{lU}+\upsilon_{y}^{lU}+\upsilon_{y}^{lU}\pi_{y}^{lL})| \end{array}$$

$$\begin{array}{ccc} & & =|(\omega_{x}^{lL}+\omega_{x}^{lL}\pi_{x}^{lU}-\upsilon_{x}^{lL}-\upsilon_{x}^{lL}\pi_{x}^{lU}+\omega_{x}^{lU}+\omega_{x}^{lU}\pi_{x}^{lL}-\upsilon_{x}^{lL}-\upsilon_{x}^{lL}\pi_{x}^{lL})-(\omega_{y}^{lL}+\omega_{y}^{lL}\pi_{y}^{lU}-\upsilon_{y}^{lL}-\upsilon_{y}^{lL}\pi_{y}^{lU}+\omega_{y}^{lU}+\omega_{y}^{lU}\pi_{y}^{lL}-\upsilon_{y}^{lL}-\upsilon_{y}^{lL}\pi_{y}^{lL})|\\ & & +|(\omega_{x}^{lL}+\omega_{x}^{lL}\pi_{x}^{lU}+\omega_{x}^{lU}+\omega_{x}^{lU}\pi_{x}^{lL})-(\omega_{y}^{lL}+\omega_{y}^{lL}\pi_{y}^{lU}+\omega_{y}^{lU}+\omega_{y}^{lU}\pi_{y}^{lL})|\\ & & +|(\upsilon_{x}^{lL}+\upsilon_{x}^{lL}\pi_{x}^{lU}+\upsilon_{x}^{lU}+\upsilon_{x}^{lU}\pi_{x}^{lL})-(\upsilon_{y}^{lL}+\upsilon_{y}^{lL}\pi_{y}^{lU}+\upsilon_{y}^{lU}+\upsilon_{y}^{lU}\pi_{y}^{lL})| \end{array}$$

$$\begin{array}{ccc} & & \leq |\omega_{x}^{lL}\left|+\right|\omega_{x}^{lL}\pi_{x}^{lU}\left|+\right|\upsilon_{x}^{lL}\left|+\right|\upsilon_{x}^{lL}\pi_{x}^{lU}\left|+\right|\omega_{x}^{lU}\left|+\right|\omega_{x}^{lU}\pi_{x}^{lL}\left|+\right|\upsilon_{x}^{lL}\left|+\right|\upsilon_{x}^{lL}\pi_{x}^{lL}\left|+\right|\omega_{y}^{lL}\left|+\right|\omega_{y}^{lL}\pi_{y}^{lU}\left|+\right|\upsilon_{y}^{lL}\left|+\right|\upsilon_{y}^{lL}\pi_{y}^{lU}\left|+\right|\omega_{y}^{lU}|\\ & & +|\omega_{y}^{lU}\pi_{y}^{lL}\left|+\right|\upsilon_{y}^{lL}\left|+\right|\upsilon_{y}^{lL}\pi_{y}^{lL}\left|+\right|\omega_{x}^{lL}\left|+\right|\omega_{x}^{lL}\pi_{x}^{lU}\left|+\right|\omega_{x}^{lU}\left|+\right|\omega_{x}^{lU}\pi_{x}^{lL}\left|+\right|\omega_{y}^{lL}\left|+\right|\omega_{y}^{lL}\pi_{y}^{lU}\left|+\right|\omega_{y}^{lU}\left|+\right|\omega_{y}^{lU}\pi_{y}^{lL}\left|+\right|\upsilon_{x}^{lL}\left|+\right|\upsilon_{x}^{lL}\pi_{x}^{lU}|\\ & & +|\upsilon_{x}^{lU}\left|+\right|\upsilon_{x}^{lU}\pi_{x}^{lL}\left|+\right|\upsilon_{y}^{lL}\left|+\right|\upsilon_{y}^{lL}\pi_{y}^{lU}\left|+\right|\upsilon_{y}^{lU}\left|+\right|\upsilon_{y}^{lU}\pi_{y}^{lL}| \end{array}$$

$$\begin{array}{ccc} & & \leq 2|\omega_{x}^{lL}+\upsilon_{x}^{lL}\left|+2\right|\omega_{x}^{lU}+\upsilon_{x}^{lU}\left|+2\right|\omega_{y}^{lL}+\upsilon_{y}^{lL}\left|+2\right|\omega_{y}^{lU}+\upsilon_{y}^{lU}|\leq 8\\ & & \text{So},\zeta_{xy}^{l}\leq 1. \end{array}$$

And,

$$2|\omega_{x}^{lL}\pi_{x}^{lU}\left|+2\right|\upsilon_{x}^{lL}\pi_{x}^{lU}\left|+2\right|\omega_{x}^{lU}\pi_{x}^{lL}\left|+2\right|\upsilon_{x}^{lL}\pi_{x}^{lL}\left|+2\right|\omega_{y}^{lL}\pi_{y}^{lU}\left|+2\right|\upsilon_{y}^{lL}\pi_{y}^{lU}\left|+2\right|\omega_{y}^{lU}\pi_{y}^{lL}\left|+2\right|\upsilon_{y}^{lL}\pi_{y}^{lL}|=0.$$

as,

$$\begin{array}{ccc} & & 2|\left(\omega_{x}^{lL}\right)\left(1-\omega_{x}^{lL}-\upsilon_{x}^{lL}\right)\left|+2\right|\left(\upsilon_{x}^{lL}\right)\left(1-\omega_{x}^{lL}-\upsilon_{x}^{lL}\right)\left|+2\right|\left(\omega_{x}^{lU}\right)\left(1-\omega_{x}^{lU}-\upsilon_{x}^{lU}\right)\left|+2\right|\left(\upsilon_{x}^{lU}\right)\left(1-\omega_{x}^{lU}-\upsilon_{x}^{lU}\right)\left|+2\right|\left(\omega_{y}^{lL}\right)\left(1-\omega_{y}^{lL}-\upsilon_{y}^{lL}\right)|\\ & & +2|\left(\upsilon_{y}^{lL}\right)\left(1-\omega_{y}^{lL}-\upsilon_{y}^{lL}\right)\left|+2\right|\left(\omega_{y}^{lU}\right)\left(1-\omega_{y}^{lU}-\upsilon_{y}^{lU}\right)\left|+2\right|\left(\upsilon_{y}^{lU}\right)\left(1-\omega_{y}^{lU}-\upsilon_{y}^{lU}\right)| \end{array}$$

$$\begin{array}{ccc} & & =2|\omega_{x}^{lL}-{\left(\omega_{x}^{lL}\right)}^{2}-\omega_{x}^{lL}\upsilon_{x}^{lL}\left|+2\right|\upsilon_{x}^{lL}-{\left(\upsilon_{x}^{lL}\right)}^{2}-\omega_{x}^{lL}\upsilon_{x}^{lL}\left|+2\right|\omega_{x}^{lU}-{\left(\omega_{x}^{lU}\right)}^{2}-\omega_{x}^{lU}\upsilon_{x}^{lU}\left|+2\right|\upsilon_{x}^{lU}-{\left(\upsilon_{x}^{lU}\right)}^{2}-\omega_{x}^{lU}\upsilon_{x}^{lU}|\\ & & +2|\omega_{y}^{lL}-{\left(\omega_{y}^{lL}\right)}^{2}-\omega_{y}^{lL}\upsilon_{y}^{lL}\left|+2\right|\upsilon_{y}^{lL}-{\left(\upsilon_{y}^{lL}\right)}^{2}-\omega_{y}^{lL}\upsilon_{y}^{lL}\left|+2\right|\omega_{y}^{lU}-{\left(\omega_{y}^{lU}\right)}^{2}-\omega_{y}^{lU}\upsilon_{y}^{lU}\left|+2\right|\upsilon_{y}^{lU}-{\left(\upsilon_{y}^{lU}\right)}^{2}-\omega_{y}^{lU}\upsilon_{y}^{lU}| \end{array}$$

$$\begin{array}{ccc} & & \leq 2|\omega_{x}^{lL}+\upsilon_{x}^{lL}\left|-2\right|{\left(\omega_{x}^{lL}\right)}^{2}+{\left(\upsilon_{x}^{lL}\right)}^{2}+2\omega_{x}^{lL}\upsilon_{x}^{lL}\left|+2\right|\omega_{x}^{lU}+\upsilon_{x}^{lU}\left|-2\right|{\left(\omega_{x}^{lU}\right)}^{2}+{\left(\upsilon_{x}^{lU}\right)}^{2}+2\omega_{x}^{lU}\upsilon_{x}^{lU}\left|+2\right|\omega_{y}^{lL}+\upsilon_{y}^{lL}|\\ & & +2|\omega_{y}^{lU}+\upsilon_{y}^{lU}\left|-2\right|{\left(\omega_{y}^{lL}\right)}^{2}+{\left(\upsilon_{y}^{lL}\right)}^{2}+2\omega_{y}^{lL}\upsilon_{y}^{lL}\left|-2\right|{\left(\omega_{y}^{lU}\right)}^{2}+{\left(\upsilon_{y}^{lU}\right)}^{2}+2\omega_{y}^{lU}\upsilon_{y}^{lU}| \end{array}$$

$$\begin{array}{ccc} & & =2-2|{\left(\omega_{x}^{lL}+\upsilon_{x}^{lL}\right)}^{2}\left|+2-2\right|{\left(\omega_{x}^{lU}+\upsilon_{x}^{lU}\right)}^{2}\left|+2+2-2\right|{\left(\omega_{y}^{lL}+\upsilon_{y}^{lL}\right)}^{2}\left|-2\right|{\left(\omega_{y}^{lU}+\upsilon_{y}^{lU}\right)}^{2}|\\ & & =2-2+2-2+2+2-2-2=0.\\ & & \text{So},\zeta_{xy}^{l}=0. \end{array}$$

 □

Property 2 $\zeta_{xy}^{l}=0,\;\zeta_{xy}^{A}=0\Leftrightarrow x=y$*and,*$\zeta_{xy}^{l}=1,\;\zeta_{xy}^{A}=1\Leftrightarrow x=-y.$

Proof $$\zeta_{xy}^{l}=\frac{1}{8}\left(\right|S_{x}^{l}\left(a_{x}\right)-S_{y}^{l}\left(a_{y}\right)\left|+\right|\Delta_{x}^{l}\left(a_{x}\right)-\Delta_{y}^{l}\left(a_{y}\right)\left|+\right|\rho_{x}^{l}\left(a_{x}\right)-\rho_{y}^{l}\left(a_{y}\right)|)=0;$$

and from support function,

$$0\leq |S_{x}^{l}\left(a_{x}\right)-S_{y}^{l}\left(a_{y}\right)\left|,0\leq \right|\Delta_{x}^{l}\left(a_{x}\right)-\Delta_{y}^{l}\left(a_{y}\right)\left|,0\leq \right|\rho_{x}^{l}\left(a_{x}\right)-\rho_{y}^{l}\left(a_{y}\right)|$$

So, from above two conditions only possibility is,

$$|S_{x}^{l}\left(a_{x}\right)-S_{y}^{l}\left(a_{y}\right)\left|=0,\right|\Delta_{x}^{l}\left(a_{x}\right)-\Delta_{y}^{l}\left(a_{y}\right)\left|=0,\right|\rho_{x}^{l}\left(a_{x}\right)-\rho_{y}^{l}\left(a_{y}\right)|=0$$

and this occurs whenever $a_{x}=a_{y}\;i.e.\;x=y.$ and hence $\zeta_{xy}^{A}=0.$

On the similar terms we can show $\zeta_{xy}^{l}=1,\;\zeta_{xy}^{A}=1\Leftrightarrow x=-y.$ □

Property 2 suggests that, in the case of two players, there will be no conflict if they both have the same perspective on the conflict problem. And when they have a different perspective on the conflict problem (when one player is entirely against the whole issue, another one is in support), the value of their conflict is 1.

Property 3 $\;\;\zeta_{xy}^{l}=\zeta_{yx}^{l}$*,*$\;\;\;\zeta_{xy}^{A}=\zeta_{yx}^{A}.$

Proof From the Eq. (7) it is quite easy to prove, as ∀x,y∈X, $\forall \;a_{l}\in A$, we have $\zeta_{xy}^{l}=\zeta_{yx}^{l}$ and we know $\zeta_{xy}=\sum_{l=1}^{p}w_{l}\zeta_{xy}^{l},$ hence, $\zeta_{xy}^{A}=\zeta_{yx}^{A}.$ □

Property 4 *Triangle inequality is obvious.*

## IVIFIS based conflict analysis system

This section put forward a new interval-valued intuitionistic fuzzy conflict analysis system with the proposed conflict distance measure’s help amid the players in an interval-valued intuitionistic fuzzy environment. Then, we offer the mathematical validation of the proposed conflict analysis system by satisfying the distance measures’ properties. From def. 6, an equivalence class defines a rough set, and two standard sets can approximate these equivalence classes, which are upper and lower approximations. The lower approximation includes all the data tuples based on the certainty of belongingness to that class without the set’s vagueness. In contrast, the set’s upper approximation involves all the data tuples based on whether data cannot be described or is not belonging to that class. Therefore, this type of uncertainty has three regions in an RS. It consists of a positive region, a negative region, and a boundary region. This subset is a rough set if the lower approximation and the upper approximation of a universal set subset are unequal. However, if lower and upper approximations are similar, the subset is a crisp set.

According to Pawlak’s system of conflict analysis, S=(X,A,Y,f) is known as the conflict information system, in which $X=\{X_{1},X_{2},\ldots ,X_{q}\}$ stands for the set of players, A denotes the conflict issue set. At the same time, Y indicates the value range of X concerning the attribute set A so that $Y_{x}^{a}$ is the value of the object $X_{x}$ concerning the conflict issue a and confined to only three values {−1,0,1} which means favorable, neutral and against. f:X×A→Y denotes the conflict information function. In the Pawlak conflict analysis rough set theory system, the players have only three attitudes concerning concern. This conflict information system developed upon the rough set was very rigid for describing and representing real-life problems involving conflicts. As a consequence of that, the system holds inconsistency with the difficulties of real conflict. We employ IVIFRS to ease the players’ attitudes over these issues. The new interval-valued intuitionistic fuzzy conflict information system (IVIFCIS) follows the real circumstances of concern, and the application framework uses the concepts of IVIFRS.

Definition 7 Given with IVIFIS Q=(X,A∪D,Y,f) and the condition attribute set $A=\left\{a_{1},a_{2},\cdots ,a_{p}\right\}$ which denotes the conflict issue set, the IVIFIS B is termed as the interval-valued intuitionistic fuzzy conflict information system (IVIFCIS). The IVIFRS over conflict distance measure is termed as the interval-valued intuitionistic fuzzy conflict analysis (IVIFCA) system.

Definition 8 With IVIFCIS, for ∀x,y∈X, $\forall a_{l}\in A$ the conflict distance $\zeta_{xy}^{l}$ is called the conflict coefficient of x,y concerning the conflict issue $a_{l}$, and conflict degree of the conflict issue $a_{l}$. The player x concerning conflict system and conflict issue set A can be respectively formalize:

(8) $$\zeta^{l}=\frac{\sum_{x,y=1,x\neq y}^{q}\zeta_{xy}^{l}}{q\left(q-1\right)}=\frac{\sum_{y=1,y\neq x}^{q}\sum_{x=1,x\neq y}^{q}\zeta_{xy}^{ml}}{q\left(q-1\right)},$$

(9) $$\zeta_{x}^{A}=\frac{\sum_{y=1,x\neq y}^{q}\zeta_{xy}^{A}}{\left(q-1\right)}=\frac{\sum_{y=1,x\neq y}^{q}\sum_{l=1}^{p}\zeta_{xy}^{l}}{p\left(q-1\right)},$$

(10) $$\zeta =\frac{\sum_{l=1}^{p}\zeta^{l}}{p}=\frac{\sum_{l=1}^{p}\sum_{x,y=1,y\neq x}^{q}\zeta_{xy}^{l}}{pq\left(q-1\right)}=\frac{\sum_{l=1}^{p}\sum_{y=1,y\neq x}^{q}\sum_{x=1,x\neq y}^{q}\zeta_{xy}^{l}}{pq\left(q-1\right)}$$

with, $x,y=1,2,\cdots ,q,x\neq y;l=1,2,\cdots ,p,\;\;\zeta_{xy}^{A},\zeta_{xy}^{l}$ respectively represent the conflict degree of x, y concerning the conflict issue set A and the conflict issue $a_{l}$. With the help of this, we can estimate the total conflict degree of the IVIFCIS, as these values calculate the conflict among the players.

In the proposed IVIFCIS, it is evident that every decision-maker values differently at every conflict problem; consequently, every conflicting issue has different weights. So, with the addition of values of decision-makers and using the definitions mentioned above, conflict degree with weights vectors is rewritten as:

Definition 9 Suppose the weight vector of the conflict issue set A is $w_{l}=\left\{w_{1},w_{2},\cdots ,w_{p}\right\}$, for ∀x,y∈X, (x,y=1,2,⋯,q,x≠y), $\forall a_{l}\in A$, the conflict degree of the player x and the conflict system concerning the conflict issue set A can be respectively formalize as:

(11) $$\zeta_{x}^{A}=\frac{\sum_{y=1,y\neq x}^{q}\zeta_{xy}^{A}}{q-1}=\frac{\sum_{y=1,y\neq x}^{q}\sum_{l=1}^{p}w_{l}\zeta_{xy}^{l}}{q-1}$$

(12) $$\zeta =\sum_{l=1}^{p}w_{l}\zeta^{l}=\frac{\sum_{l=1}^{p}\sum_{y=1,y\neq x}^{q}w_{l}\zeta_{xy}^{l}}{q\left(q-1\right)}$$

where $w_{l}$ denotes the player’s weight for the conflict issue $a_{l}\left(l=1,2,\cdots ,p\right)$, assigned by the decision makers with $\sum_{l=1}^{p}w_{l}=1$.

### Coalitions for conflict analysis system over IVIFRS

For the organic analysis and the conflict analysis system’s definition, the ongoing paper discusses the player’s attitude, the issues, or options to take on conflict attribute values. It will allow the player to make any subtle changes for conflict evolution by embracing several options to control players’ conflict degrees. So the players are allowed to use several different options about various conflict issues provided that the conflict degrees of conflict information are highly significant. It will minimize the conflict degrees of the information system so that the system can have stability. Thus, there should be a coalition for the system to be stable and reduce the system’s conflict. So coalition for conflict analysis system over IVIFRS over the threshold value is given below:

Definition 10 For the conflict issue set A, U⊆X, ∀x,y∈U, γ∈[0,1], the coalition $U^{\gamma }$ established on the threshold value γ is:

(13) $$U^{\gamma }=\left\{x\in U|\;\zeta_{xy}^{A}\leq \gamma ,\forall x,y\in U\right\}$$

If $\zeta_{xy}^{A}>\gamma$, then there is no coalition amidst the players.

Property 5 *For*γ∈[0,1]*,*$U^{\gamma }\subseteq U$*and*$U^{\gamma }=U$*only when*γ=1*.*

Proof With the help of Definition 10, proof is obvious. □

## Rough set

Definition 11 For a given IVIFIS, Q=(X,A∪D,Y,f) a threshold value Λ∈[0,1] and attribute set $T=\left(t_{1},t_{2},\cdots ,t_{l}\right)$, T⊆A, U⊆X, suppose that the Λ−T neighborhood bases of x is ${\left[x\right]}_{\Lambda }^{T}=\left\{y\in X|\;\xi_{yx}^{t}\leq \Lambda ,\forall \;t\in T\right\}$. Then the (Λ−T) - lower approximation and (Λ−T) - upper approximation of U are respectively given as:

(14) $${\underline{apr}}_{T}^{\Lambda }\left(U\right)=\cup \left\{x\in X|{\left[x\right]}_{T}^{\Lambda }\subseteq U\right\}$$

(15) $${\bar{apr}}_{T}^{\Lambda }\left(U\right)=\cup \left\{x\in X|{\left[x\right]}_{T}^{\Lambda }\cap U\neq \phi \right\}$$

As per the def. 11, setting the threshold Λ, and the acquired ${\left[x\right]}_{T}^{\Lambda }$, the (Λ−T)- lower approximation and the (Λ−T) - upper approximation of U are obtained. The proposed system can be considered an interval-valued intuitionistic fuzzy rough set (IVIFRS) system. The (Λ−T)- lower approximation ${\underline{apr}}_{T}^{\Lambda }\left(U\right)$ of the set U can also be termed the positive region of the IVIFRS system based on novel conflict distance.

For the given confidence threshold values Λ, ${\underline{apr}}_{T}^{\Lambda }\left(U\right)$ is such a set that positively belongs to the set U under the universe X. The (Λ−T)- upper approximation ${\bar{apr}}_{T}^{\Lambda }\left(U\right)$ of the set is such a set that possibly belongs to set U, where x considers the given threshold values Λ under the universe X. The upper approximation and lower approximations of the model is used to interpret the (Λ−T) boundary, classification quality and approximation accuracy of U, which is defined below:

Theorem 1 *For a given IVIFIS*Q=(X,A∪D,Y,f)*,*0≤Λ≤1*, the*(Λ−T)*boundary, approximation accuracy and classification quality of*U*could be defined as:*

$$boundary_{T}^{\Lambda }\left(U\right)={\bar{apr}}_{T}^{\Lambda }\left(U\right)-{\underline{apr}}_{T}^{\Lambda }\left(U\right)$$

$$\tilde{\alpha }\left(U\right)=\frac{\left|{\underline{apr}}_{T}^{\Lambda }\left(U\right)\right|}{\left|{\bar{apr}}_{T}^{\Lambda }\left(U\right)\right|}$$

$$\gamma^{\Lambda }\left(T,D\right)=\frac{\left|{\underline{apr}}_{T}^{\Lambda }\left(U\right)\right|}{\left|X\right|}$$

Proof Proof is obvious. □

Theorem 2 ${\underline{apr}}_{T}^{\Lambda }\left(U\right)\subseteq U\subseteq {\bar{apr}}_{T}^{\Lambda }\left(U\right).$

Proof Let $x\in {\underline{apr}}_{T}^{\Lambda }\left(U\right)$. Then there exist ${\left[x\right]}_{T}^{\Lambda }\subseteq U$ and $x\in {\left[x\right]}_{T}^{\Lambda }$ so that x∈U Therefore, ${\underline{apr}}_{T}^{\Lambda }\left(U\right)\subseteq U$. From x∈U, it follows that ${\left[x\right]}_{T}^{\Lambda }\cap U\neq \phi$. Thus, we get $x\in {\bar{apr}}_{T}^{\Lambda }\left(U\right)$. Therefore, $U\subseteq {\bar{apr}}_{T}^{\Lambda }\left(U\right)$. □

Theorem 3 *For a given IVIFIS*Q=(X,A∪D,Y,f)*,*U∈X*,*$T_{1}\subseteq T_{2}\subseteq A$0≤Λ≤1*, then the following holds true:*

$\left(1\right)\;{\underline{apr}}_{T_{2}}^{\Lambda }\left(U\right)\subseteq {\underline{apr}}_{T_{1}}^{\Lambda }\left(U\right)$*, and*

$\left(2\right)\;{\bar{apr}}_{T_{2}}^{\Lambda }\left(U\right)\subseteq {\bar{apr}}_{T_{1}}^{\Lambda }\left(U\right)$

Proof (1) For $\forall x\in {\underline{apr}}_{T_{1}}^{\Lambda }\left(U\right)$, there exists ${\left[x\right]}_{T_{1}}^{\Lambda }\subseteq U$. From $T_{1}\subseteq T_{2}\subseteq A$ we have ${\left[x\right]}_{T_{2}}^{\Lambda }\subseteq {\left[x\right]}_{T_{1}}^{\Lambda }$. Thus $x\in {\underline{apr}}_{T_{1}}^{\Lambda }\left(U\right)$. Therefore, ${\underline{apr}}_{T_{2}}^{\Lambda }\left(U\right)\subseteq {\underline{apr}}_{T_{1}}^{\Lambda }\left(U\right)$.

(2) For $\forall x\in {\underline{apr}}_{T_{2}}^{\Lambda }\left(U\right)$, there exists ${\left[y\right]}_{T_{2}}^{\Lambda }\cap U\neq \phi$.From $T_{1}\subseteq T_{2}\subseteq A$, we have ${\left[y\right]}_{T_{1}}^{\Lambda }\cap U\neq \phi$.Thus $y\in {\bar{apr}}_{T_{1}}^{\Lambda }\left(U\right)$. Therefore, ${\bar{apr}}_{T_{2}}^{\Lambda }\left(U\right)\subseteq {\bar{apr}}_{T_{1}}^{\Lambda }\left(U\right)$. □

Theorem 4 *For a given IVIFIS*Q=(X,A∪D,Y,f)*,*U⊆X*,*T⊆A*,*$0\leq \lambda_{1}\leq \lambda_{2}\leq 1$*, the following hold true:*

$\left(1\right)\;{\underline{apr}}_{T}^{\Lambda_{2}}\left(U\right)\subseteq {\underline{apr}}_{T}^{\Lambda_{1}}\left(U\right)$

$\left(2\right)\;{\bar{apr}}_{T}^{\Lambda_{1}}\left(U\right)\subseteq {\bar{apr}}_{T}^{\Lambda_{2}}\left(U\right)$

Proof (1) For $\forall x\in {\underline{apr}}_{T}^{\Lambda_{2}}\left(U\right)$, there exists ${\left[x\right]}_{T}^{\Lambda_{2}}\subseteq U$. because of $0\leq \Lambda_{1}\leq \Lambda_{2}\leq 1$ we get ${\left[x\right]}_{T}^{\Lambda_{1}}\subseteq {\left[x\right]}_{T}^{\Lambda_{2}}$. Thus $x\in {\underline{apr}}_{T}^{\Lambda_{1}}\left(U\right)$. Therefore, ${\underline{apr}}_{T}^{\Lambda_{2}}\left(U\right)\subseteq {\underline{apr}}_{T}^{\Lambda_{1}}\left(U\right)$.

(2) For $\forall y\in {\bar{apr}}_{T}^{\Lambda_{1}}\left(U\right)$, there exists ${\left[y\right]}_{T}^{\Lambda_{1}}\cap U\neq \phi$.so that we have ${\left[y\right]}_{T}^{\Lambda_{2}}\cap U\neq \phi$ so that.Thus $y\in {\bar{apr}}_{T}^{\Lambda_{2}}\left(U\right)$. Therefore, ${\bar{apr}}_{T}^{\Lambda_{1}}\left(U\right)\subseteq {\bar{apr}}_{T}^{\Lambda_{2}}\left(U\right)$. □

Remark 3 If $\omega_{A}^{L}\left(x\right)=\omega_{A}^{U}\left(x\right)$ and $\upsilon_{A}^{L}\left(x\right)=\upsilon_{A}^{U}\left(x\right)$ then the above mathematical results are valid for AIFS domain.

## Case study

This section illustrates numerical examples of decision-making problems with conflict interests to calculate the efficacy and accuracy of the suggested model.

**Conversion scale of IFS data of** [40] **into IVIFS data:**

Let (ω,υ) be the membership and non-membership value of a given AIFS data. Then its conversion into IVIFS data is given below:

Membership interval $\left(M^{\prime })=[1-\omega ,\omega \right]$, and non-membership interval $\left(N^{\prime })=[0,\upsilon \right]$, then, $\;([\omega_{1}^{L},\omega_{1}^{U}],[\upsilon_{1}^{L},\upsilon_{1}^{U}])=([1-\omega ,\omega ],[0,\upsilon ])$

Example 1 After the conversion of IFS data adapted from Liu and Lin [40] to IVIFS data, we get the Table 1. The collaboration of the University and industry is a thoughtful way to modernism. Since the conflict problem affects and hinders collaboration accomplishment, it is essential to study university and industry collaboration conflict problems. The issue of conflict between the University and industry collaboration primarily arises from the different -different interests, which is shown with three conditions, intellectual property rights (marked as $a_{1}$), administration authority (marked as $a_{2}$), knowledge gains (marked as $a_{3}$). The collaboration of the University and industry connects three parties, namely the pedigrees of knowledge (research institutes, universities represented by $X_{1}$), the beneficiaries of knowledge (enterprises, represented by $X_{2}$), and the supervisor (mediator of science and technology, the Government, expressed by $X_{3}$). To obtain the relevant data involving conflicts, a panel of 30 professionals (10 professionals from the University, ten professionals from enterprises, and ten from the Government) will conduct the cross-examinations. The IVIFIS on University and industry conflict collaboration is acquired and expressed by compiling and categorizing the data in Table 1.

**Table 1:** IVIFIS on collaboration of university and industry conflict.

	$a_{1}$	$a_{2}$	$a_{3}$
$\;\;\;X_{1}$	([0.2,0.8],[0.0,0.0])	([0.0,0.0],[0.0,0.5])	([0.4,0.6],[0.0,0.0])
$\;\;\;X_{2}$	([0.0,0.0],[0.0,0.6])	([0.2,0.8],[0.0,0.0])	([0.0,0.0],[0.0,0.7])
$\;\;\;X_{3}$	([0.4,0.6],[0.0,0.0])	([0.3,0.7],[0.0,0.1])	([0.3,0.7],[0.0,0.0])

After calculating the conflict distances between the three parties and three aspects, results are shown in Table 2, we can see that the conflict distance between the pedigrees of knowledge ($X_{1}$) and the beneficiaries of knowledge ($X_{2}$) concerning knowledge gain ($a_{3}$) is the maximum (0.5975), and these values are more relatable and consistent to the factual circumstances.

**Table 2:** Conflict distance for cooperation of university and industry in IVIFIS.

	a=1			a=2			a=3		
	$\;\;X_{1}$	$\;\;X_{2}$	$\;\;X_{3}$	$\;\;X_{1}$	$\;\;X_{2}$	$\;\;X_{3}$	$\;\;X_{1}$	$\;\;X_{2}$	$\;\;X_{3}$
$\;\;X_{1}$	0	0.5400	0.0400	0	0.5175	0.4950	0	0.5975	0.0150
$\;\;X_{2}$	0.5400	0	0.5800	0.5175	0	0.0300	0.5975	0	0.5825
$\;\;X_{3}$	0.0400	0.5800	0	0.4950	0.0300	0	0.0150	0.5825	0

Conflict degree of conflict issues $a_{l}$ are calculated with Eq. (8) by taking the value of q=3 in IVIFCIS which is given as:

$$\zeta^{1}=0.3866,\;\zeta^{2}=0.3475,\;\zeta^{3}=0.3983.$$

where, $\zeta^{1},\zeta^{2},\zeta^{3}$ are conflict degrees of conflict issues $a_{1},a_{2},a_{3}$ respectively of players concerning the conflict system.

Conflict degree of conflict issue set A are calculated with Eq. (9) by taking the value of p=q=3 in IVIFCIS which is given as:

$$\;\zeta_{1}^{A}=0.3675,\;\zeta_{2}^{A}=0.4745,\;\zeta_{3}^{A}=0.2904.$$

where, $\zeta_{1}^{A},\zeta_{2}^{A},\zeta_{3}^{A}$ are conflict degrees of conflict issue set A with respect to the players $X_{1},X_{2},X_{3}$ respectively in conflict system.

Total conflict degree of the IVIFCIS is calculated using the Eq. (10),

ζ=0.3774.

Using the weights given by the decision makers say (0.37,0.28,0.35), the improved conflict degree by weight vectors of conflict issue set A are calculated with Eq. (11) by taking the value of p=q=3 in IVIFCIS which is given by,

$$\zeta_{1}^{A}=0.3500,\;\zeta_{2}^{A}=0.4903,\zeta_{3}^{A}=0.2928.$$

where, $\zeta_{1}^{A},\zeta_{2}^{A},\zeta_{3}^{A}$ are conflict degrees of conflict issue set A with respect to the players $X_{1},X_{2},X_{3}$ respectively in conflict system.

Then the improved total conflict degree of the IVIFCIS with weight vectors, which is given with the help of Eq. (12):

ζ=0.3797.

For γ≤0.52, the coalition among the pedigrees of knowledge $\left(X_{1}\right)$ and the coordinator $\left(X_{3}\right)$ concerning the conflict issues of intellectual property rights $\left(a_{1}\right)$, administration authority $\left(a_{2}\right)$ and knowledge gains $\left(a_{3}\right)$ is found. The coalition among the beneficiaries of knowledge $\left(X_{2}\right)$ and the supervisor $\left(X_{3}\right)$ concerning the conflict issue of only administration authority $\left(a_{2}\right)$ is found. There is no coalition among them with conflict issues of intellectual property $\left(a_{1}\right)$ and knowledge gain $\left(a_{3}\right)$. In contrast, the coalition between the pedigrees of knowledge $\left(X_{1}\right)$ and the beneficiaries of knowledge $\left(X_{2}\right)$ is not originated, which is more relatable and compatible with the factual circumstances.

With the choice of γ≤0.63 [40], there will be a complete coalition between the university-industry cooperation conflict, which is seldom the case. Various alliances are formed by changing the value of γ as per our need. The proposed system reduces the system’s conflict from the total conflict in [40], which is suitable for a proper and long-lasting coalition between them.

Example 2 After the conversion of IFS data adapted from Liu and Lin [40] to IVIFS data, we get the Table 3. Economic improvement and social balance are essential jobs of the local government in China. So, Henan province has pointed out many matters for reforming the economy and administrating the differences amongst the seven constituencies: building/road construction, job, electricity problem, educational institutions, and transport system; to figure out and solve their rising conflict problems. Seven constituencies, namely Pingyu, Runan, Shangcai, Queshan, Xincai, Xiping, and, Suiping, can be respectively thought of as the set of players and recorded as $X_{1}$, $X_{2}$, $X_{3}$, $X_{4}$, $X_{5}$, $X_{6}$, $X_{7}$ and the issues are building/road construction, job, electricity problem, educational institutions, and transport system are shown as $a_{1}$, $a_{2}$, $a_{3}$, $a_{4}$, $a_{5}$ and the conflict problem can be seen in IVIFCIS. For the real-world decision-making conflict information system, because of its vagueness and intricacy and the different opinion of decision-makers, the players’ conflict values for conflict matters are given in IVIFNs. The situation of seven constituencies concerning the five conflict matters accounted for and investigated by the Henan province government in 2013 urged 70 delegates in which every constituency has ten members, to suggest their needs and approach for five conflict matters, With an IVIFCIS= (X,A∪D,Y,f), whose details are shown in Table 3, with X={$X_{1}$, $X_{2}$, $X_{3}$, $X_{4}$, $X_{5}$, $X_{6}$, $X_{7}$} is the set of players, A = {$a_{1}$, $a_{2}$, $a_{3}$, $a_{4}$, $a_{5}$} be the set of conflict issues and Y the range for the conflict values of players concerning the conflict matters and d stands for the decision attribute with full approval and disapproval denoted by 1, 2 respectively.

**Table 3:** Interval valued intuitionistic fuzzy conflict system.

	$\;\;\;\;\;\;\;\;\;\;a_{1}$	$\;\;\;\;\;\;\;\;\;\;a_{2}$	$\;\;\;\;\;\;\;\;\;\;a_{3}$	$\;\;\;\;\;\;\;\;\;\;a_{4}$	$\;\;\;\;\;\;\;\;\;\;a_{5}$	d
$X_{1}$	([0.4,0.6],[0.0,0.3])	([0.2,0.8],[0.0,0.1])	([0.3,0.7],[0.0,0.3])	([0.1,0.9],[0.0,0.1])	([0.2,0.8],[0.0,0.2])	1
$X_{2}$	([0.3,0.7],[0.0,0.3])	([0.2,0.8],[0.0,0.2])	([0.4,0.6],[0.0,0.4])	([0.3,0.7],[0.0,0.3])	([0.2,0.8],[0.0,0.2])	1
$X_{3}$	([0.3,0.7],[0.0,0.3])	([0.5,0.5],[0.0,0.2])	([0.4,0.6],[0.0,0.4])	([0.4,0.6],[0.0,0.4])	([0.2,0.8],[0.0,0.2])	2
$X_{4}$	([0.3,0.7],[0.0,0.3])	([0.4,0.6],[0.0,0.2])	([0.3,0.7],[0.0,0.3])	([0.2,0.8],[0.0,0.2])	([0.2,0.8],[0.0,0.1])	1
$X_{5}$	([0.3,0.7],[0.0,0.3])	([0.5,0.5],[0.0,0.1])	([0.3,0.7],[0.0,0.3])	([0.4,0.6],[0.0,0.4])	([0.2,0.8],[0.0,0.2])	1
$X_{6}$	([0.4,0.6],[0.0,0.2])	([0.5,0.5],[0.0,0.1])	([0.4,0.6],[0.0,0.4])	([0.3,0.7],[0.0,0.3])	([0.4,0.6],[0.0,0.2])	2
$X_{7}$	([0.4,0.6],[0.0,0.4])	([0.3,0.7],[0.0,0.1])	([0.5,0.5],[0.0,0.3])	([0.3,0.7],[0.0,0.1])	([0.4,0.6],[0.0,0.2])	2

The value ([0.4,0.6],[0.0,0.3]) of $X_{1}$ in Table 3 for the conflict matter $a_{1}$ tells us that from 40 to 60 percent delegates from Pingyu reasonably approve to appeal to Zhumandian city for road construction, at the same time 0 to 30 percent delegates disapproving the same, and 10 to 40 percent delegates are inactive.

The new conflict distance (see: Eq. (7)) between the players corresponding to the set of conflict matters are shown below in Table 4, Table 5, Table 6.

**Table 4:** Conflict distance for $a_{1}$ and $a_{2}$.

	a=1							a=2						
	$X_{1}$	$X_{2}$	$X_{3}$	$X_{4}$	$X_{5}$	$X_{6}$	$X_{7}$	$X_{1}$	$X_{2}$	$X_{3}$	$X_{4}$	$X_{5}$	$X_{6}$	$X_{7}$
$X_{1}$	0	0.0225	0.0225	0.0225	0.0225	0.0375	0.0375	0	0.0425	0.0400	0.0325	0.0525	0.0525	0.0275
$X_{2}$	0.0225	0	0	0	0	0.0525	0.0525	0.0425	0	0.0600	0.0500	0.0875	0.0875	0.0675
$X_{3}$	0.0225	0	0	0	0	0.0525	0.0525	0.0400	0.0600	0	0.0100	0.0425	0.0425	0.0350
$X_{4}$	0.0225	0	0	0	0	0.0525	0.0525	0.0325	0.0500	0.0100	0	0.0475	0.0475	0.0300
$X_{5}$	0.0225	0	0	0	0	0.0525	0.0250	0.0525	0.0875	0.0425	0.0475	0	0	0.0250
$X_{6}$	0.0375	0.0525	0.0525	0.0525	0.0525	0	0.0700	0.0525	0.0875	0.0425	0.0475	0	0	0.0250
$X_{7}$	0.0375	0.0525	0.0525	0.0250	0.0250	0.0700	0	0.0275	0.0675	0.0350	0.0300	0.0250	0.0250	0

**Table 5:** Conflict distance for $a_{3}$ and $a_{4}$.

	a=3							a=4						
	$X_{1}$	$X_{2}$	$X_{3}$	$X_{4}$	$X_{5}$	$X_{6}$	$X_{7}$	$X_{1}$	$X_{2}$	$X_{3}$	$X_{4}$	$X_{5}$	$X_{6}$	$X_{7}$
$X_{1}$	0	0.0250	0.0400	0	0	0.0250	0.0350	0	0.0500	0.0750	0.0250	0.0750	0.0500	0.0650
$X_{2}$	0.0250	0	0.0225	0.0250	0.0250	0	0.0375	0.0500	0	0.0250	0.0250	0.0250	0	0.0800
$X_{3}$	0.0400	0.0225	0	0.0400	0.0400	0.0225	0.0600	0.0750	0.0250	0	0.0500	0	0.0250	0.0975
$X_{4}$	0	0.0250	0.0400	0	0	0.0250	0.0350	0.0250	0.0250	0.0500	0	0.0500	0.0250	0.0675
$X_{5}$	0	0.0250	0.0400	0	0	0.0250	0.0350	0.0750	0.0250	0	0.0500	0	0.0250	0.0975
$X_{6}$	0.025	0	0.0225	0.0250	0.0250	0	0.0375	0.0500	0	0.0250	0.0250	0.0250	0	0.0800
$X_{7}$	0.0350	0.0375	0.0600	0.0350	0.0350	0.0375	0	0.0650	0.0800	0.0975	0.0675	0.0975	0.0800	0

**Table 6:** Conflict distance for $a_{5}$.

	$\;\;\;\;\;X_{1}$	$\;\;\;\;\;X_{2}$	$\;\;\;\;\;X_{3}$	$\;\;\;\;\;X_{4}$	$\;\;\;\;\;X_{5}$	$\;\;\;\;\;X_{6}$	$\;\;\;\;\;X_{7}$
$\;\;\;X_{1}$	0	0	0	0.0425	0	0.0500	0.0500
$\;\;\;X_{2}$	0	0	0	0.0425	0	0.0500	0.0500
$\;\;\;X_{3}$	0	0	0	0.0425	0	0.0500	0.0500
$\;\;\;X_{4}$	0.0425	0.0425	0.0425	0	0.0425	0.0325	0.0325
$\;\;\;X_{5}$	0	0	0	0.0425	0	0.0500	0.0500
$\;\;\;X_{6}$	0.0500	0.0500	0.0500	0.0325	0.0500	0	0
$\;\;\;X_{7}$	0.0500	0.0500	0.0500	0.0325	0.0500	0	0

Conflict degree of conflict issues $a_{l}$ are calculated with Eq. (8) by taking the value of q=5 in IVIFCIS which is estimated as:

$$\;\zeta^{1}=0.0257,\;\zeta^{2}=0.0430,\;\zeta^{3}=0.0264,\;\zeta^{4}=0.0482,\;\zeta^{5}=0.0302$$.

where, $\zeta^{1},\zeta^{2},\zeta^{3},\zeta^{4},\zeta^{5}$ are conflict degrees of conflict issues $a_{1},a_{2},a_{3},a_{4},a_{5}$ respectively of players with respect to IVIFCIS.

In the context of the current conflict problem and looking at the obtained values of conflict degrees for the conflict issues $a_{l}$, it can be seen that educational institutes are the crucial primary issue. After that, factories, railways, entertainment, and road construction are the other essential issues in the seven provinces of China.

Now to check the conflict degrees amongst the players, based upon the weight vectors [60], the weight concerning the conflict issue $a_{l}$ is respectively obtained below as:

$$\;w_{l}=\left(\;w_{1},\;w_{2},\;w_{3},\;w_{4},\;w_{5}\right)=\left(0.1516,\;0.2481,\;0.1112,\;0.3274,\;0.1617\right)$$

The improved conflict degree by weight vectors of conflict issue set A are calculated with Eq. (11), by taking the value of p=7,q=5 in IVIFCIS which is given by,

$$\begin{array}{c} \zeta_{1}^{A}=0.0389,\;\zeta_{2}^{A}=0.0364,\;\zeta_{3}^{A}=0.0349,\\ \zeta_{4}^{A}=0.0334,\;\zeta_{5}^{A}=0.0341,\;\zeta_{6}^{A}=0.0385,\;\zeta_{7}^{A}=0.0511 \end{array}$$.

where, $\zeta_{1}^{A},\zeta_{2}^{A},\zeta_{3}^{A},\zeta_{4}^{A},\zeta_{5}^{A},\zeta_{6}^{A},\zeta_{7}^{A}$ are conflict degrees of conflict issue set A with respect to the players (countries) $X_{1},X_{2},X_{3},X_{4},X_{5},X_{6},X_{7}$ respectively in conflict system.

For the IVIFCIS, the improved total conflict degree by weight vectors of the conflict information system of seven countries is determined with the help of Eq. (12) as:

ζ=0.0381.

For the given IVIFCIS, to further minimize the conflict degree and to make the system more stable, Zhumandian city may use many tricks so that the seven countries can change their attitudes and conflicting issues. For betterment and development, a coalition is formed between the countries. In this scenario, governments must form alliances to advance their people to obtain more capital from Zhumadian city. As per the computations and scrutiny of the conflict degrees obtained above, with setting the different threshold values, in Table 8, the other countries’ various coalitions concerning the conflict issues are obtained. Following Tables 7 and 8, Shangcai $\left(X_{3}\right)$ and Xincai $\left(X_{5}\right)$ are the easiest to give the coalition. On the other hand, Runan $\left(X_{2}\right)$, Shangcai $\left(X_{3}\right)$, Pingyu $\left(X_{1}\right)$ and Queshan $\left(X_{4}\right)$ are mild in partnership. From Tables 7 and 8, the degrees of the seven countries’ conflicts are different, and the diverse coalition is formed using various thresholds. The number of countries in the resulting partnership shrinks as soon as the threshold value decreases. Whenever the current discussed system drops its threshold value by **0.0150**, there is no coalition among the countries as the degree of conflict shoots up. It shoots up beyond the countries resistance of conflict value, and the IVIFCIS does not fulfill the settled threshold value. For having more coalition between the countries by analyzing Tables 7 and 8, Zhumandian city can force the seven countries to alter their attitude towards the conflict issues procure more and more coalition. The proposed system reduces the real conflict of the system from the total conflict in [40], which is healthy for the coalition.

**Table 7:** Improved conflict degrees among the players.

	$\;\;\;\;\;X_{1}$	$\;\;\;\;\;X_{2}$	$\;\;\;\;\;X_{3}$	$\;\;\;\;\;X_{4}$	$\;\;\;\;\;X_{5}$	$\;\;\;\;\;X_{6}$	$\;\;\;\;\;X_{7}$
$\;\;\;X_{1}$	0	0.0331	0.0423	0.0265	0.0410	0.0459	0.0450
$\;\;\;X_{2}$	0.0331	0	0.0256	0.0302	0.0327	0.0378	0.0590
$\;\;\;X_{3}$	0.0423	0.0256	0	0.0302	0.0150	0.0373	0.0592
$\;\;\;X_{4}$	0.0265	0.0302	0.0302	0	0.0350	0.0360	0.0425
$\;\;\;X_{5}$	0.0410	0.0327	0.0150	0.0350	0	0.0270	0.0539
$\;\;\;X_{6}$	0.0459	0.0378	0.0373	0.0360	0.0270	0	0.0472
$\;\;\;X_{7}$	0.0450	0.0590	0.0592	0.0425	0.0539	0.0472	0

**Table 8:** The coalition for different threshold values.

Threshold	Coalition
0.05	$\;\{X_{1},X_{4},X_{5},X_{6}\}$$\;\{X_{2},X_{3}\}$$\;\{X_{6},X_{7}\}$
0.04	$\;\{X_{1},X_{2},X_{4}\}$$\;\{X_{2},X_{3},X_{4},X_{5},X_{6}\}$$\;\{X_{7}\}$
0.035	$\;\{X_{1},X_{2},X_{4}\}$$\;\{X_{2},X_{3},X_{4},X_{5}\}$$\;\{X_{5},X_{6}\}$$\;\{X_{7}\}$
0.03	$\;\{X_{1},X_{4}\}$$\;\{X_{2},X_{3}\}$$\;\{X_{3},X_{5}\}$$\;\{X_{5},X_{6}\}$$\;\{X_{7}\}$
0.0275	$\;\{X_{1},X_{4}\}$$\;\{X_{2},X_{3}\}$$\;\{X_{3},X_{5}\}$$\;\{X_{5},X_{6}\}$$\;\{X_{7}\}$
0.02	$\;\{X_{1}\}$$\;\{X_{2}\}$$\;\{X_{3},X_{5}\}$$\;\{X_{4}\}$$\;\{X_{6}\}$$\;\{X_{7}\}$
0.01	$\;\{X_{1}\}$$\;\{X_{2}\}$$\;\{X_{3}\}$$\;\{X_{4}\}$$\;\{X_{5}\}$$\;\{X_{6}\}$$\;\{X_{7}\}$

With the obtained results and analysis, it is clear that the basic idea for coalition formation is how conflict distance is defined and the way threshold value is fixed. As proposed in this paper, the conflict analysis system over IVIFRS handles the earlier proposed conflict analysis system’s process on the rough set in a more simplified way. Moreover, it also reduces the system’s total conflict by using a new conflict distance based on an improved score function that can differentiate between the given data values [40]. And with reduced conflict in the system, there are more chances of a coalition between the players. To add more, this new IVIFRS based on the conflict distance system provides a better simplification process. It suggests different ways of coalition formation to meet other requirements too.

## Conclusion

The existence of conflict is inevitable in this progressive society. With the rapid ongoing globalization, the conflicts and their varieties are diverging. It is primarily needed to understand these conflicts and resolve these real-world conflicts because of the real world’s uncertainty. Real-world problems can be complex, carrying a specific sequence of events for an uncertain situation and keeping in mind human limitations. It has become tough to apply one computing technique to describe real-world conflict problems to an appreciable extent. Accepting these facts, we have realized that we should merge the advantages of different methods of uncertainty and propose a potent blend of different methodologies that will serve as a powerful hybrid tool for real-world computing conflicts. This paper discusses the novel conflict distance measure based on a new score function. Using IVIFS and RS theories together proposes an IVIFRS system based on the new conflict distance. The proposed conflict analysis system over IVIFRS is studied with the introduction of weight vectors too. The proposed system provides a better real-world uncertainty simplification process in the IVIFS domain. It reduces the system’s real conflict, as described by earlier proposed theories concerning the system’s conflict. It considers a wide range of real-world conflict problems as it considers IVIFSs, which are more supple to a real-world scenario and are underutilized.

We also wish to extend our proposed system to a dominance-based interval-valued intuitionistic fuzzy rough set using conflict distance in the near future. We also want to give a proper detailed analysis of the threshold value so that the system can attain its desired equilibrium.

## Declaration of Competing Interest

The authors declare that they have no known competing financial interests or personal relationships that could have appeared to influence the work reported in this paper.

## Data Availability

Data will be made available on request.
